# Decoding the glycoproteome: a new frontier for biomarker discovery in cancer

**DOI:** 10.1186/s13045-024-01532-x

**Published:** 2024-03-22

**Authors:** Kai He, Maryam Baniasad, Hyunwoo Kwon, Tomislav Caval, Gege Xu, Carlito Lebrilla, Daniel W. Hommes, Carolyn Bertozzi

**Affiliations:** 1 James Comprehensive Cancer Center, The Ohio State University, Columbus, USA; 2 InterVenn Biosciences, South San Francisco, USA; 3 Department of Biochemistry and Molecular Medicine, UC Davis Health, Sacramento, USA; 4 Department of Chemistry, Stanford University, Stanford, USA

**Keywords:** Glycoproteomics, Biomarker, Cancer, Screening

## Abstract

Cancer early detection and treatment response prediction continue to pose significant challenges. Cancer liquid biopsies focusing on detecting circulating tumor cells (CTCs) and DNA (ctDNA) have shown enormous potential due to their non-invasive nature and the implications in precision cancer management. Recently, liquid biopsy has been further expanded to profile glycoproteins, which are the products of post-translational modifications of proteins and play key roles in both normal and pathological processes, including cancers. The advancements in chemical and mass spectrometry-based technologies and artificial intelligence-based platforms have enabled extensive studies of cancer and organ-specific changes in glycans and glycoproteins through glycomics and glycoproteomics. Glycoproteomic analysis has emerged as a promising tool for biomarker discovery and development in early detection of cancers and prediction of treatment efficacy including response to immunotherapies. These biomarkers could play a crucial role in aiding in early intervention and personalized therapy decisions. In this review, we summarize the significant advance in cancer glycoproteomic biomarker studies and the promise and challenges in integration into clinical practice to improve cancer patient care.

## Introduction

Cancer continues to be a significant source of human suffering, with an annual toll of 9.9 million lives lost and an additional 18.1 million new cases reported each year [1]. Biomarker discovery holds a key to significant advancement in cancer prevention and treatment, with applications in risk estimation, disease screening and early detection, diagnosis, prognosis, therapeutic prediction, and disease monitoring. Cancers are heterogeneous at every level, including molecular and cellular variations, histopathology, and clinical manifestations, which makes biomarker discovery an essential need for the vision of precision medicine. Multi-omics integration, including next-generation sequencing (NGS) and comprehensive immunohistochemical (IHC) profiles, have enabled the development of targeted therapies (e.g., against EGFR, Her2/neu, ALK, BRAF, and others) and immune therapies. These personalized strategies leverage individual patients’ unique dynamic molecular and cellular changes to improve cancer survival [2].

Since the 1950s, the concept of cancer liquid biopsy involved examining blood protein biomarkers [3]. While many protein biomarkers have been developed and approved for cancer diagnosis and monitoring (e.g., PSA, CA125), improved specificity and sensitivity remain as critical challenges [4]. The modern concept of cancer liquid biopsy has shifted to the analysis of circulating tumor cells (CTCs) and circulating tumor-derived DNA (ctDNA) [5]. Highly sensitive and specific technologies based on multiplex PCR (mPCR) or next-generation sequencing (NGS) have rapidly evolved, enabling large-scale detection of genetic alterations in circulating nucleic acids, including gene mutations, fusions, deletions, amplifications, translocations, epigenetic changes, and DNA fragmentomics of ctDNA in liquid biopsy studies [6].

In addition to nucleic acid-based strategies, proteomic profiling of cancer liquid biopsies has gained significant traction [7]. Proteins directly govern normal cellular functions, and the aberrant protein expression, post-translational modification, subcellular localization, or function (caused by mutations and post-translational modifications) can drive oncogenesis and serve as targets for cancer therapies. Due to its versatility in detecting and quantitating biological molecules, mass spectrometry (MS) has superseded gel-based approaches of previous decades and is now the standard technology for proteomics analyses [8]. High-dimensional proteomic data offer unprecedented insights for biomarker discovery and clinical implementation [9]. Liquid biopsy protein profiles provide organ-specific information, surpassing DNA/RNA and aiding in tumor origin identification. Combining novel protein biomarkers with nucleic acids can significantly improve diagnostic accuracy.

Gaining insights into post-translational protein modifications substantially enhances the quantity of cancer-relevant information attainable from these proteins. Protein glycosylation, a common post-translational modification, involves attaching glycans to proteins primarily via *N*- (asparagine) or *O*- (serine/threonine) linkages [10]. It affects various physiological events, including protein folding and stability and trafficking, cell–cell interactions, differentiation, and the immune response [11]. Aberrant protein glycosylation is a hallmark of cancer, crucial in malignant transformation, tumor development, invasiveness, and metastatic disease [12–14]. It significantly influences cancer-immune system interactions, including immunosurveillance and immunoediting. Unique tumor-specific glycosylation patterns frequently manifest in the forms of increased branching of *N*-glycans, higher density of *O*-glycans, incomplete synthesis of glycans, neosynthesis, increased sialylation, and increased fucosylation [15, 16], which are promising targets for liquid biopsy for discrimination between the benign and cancerous cells. Despite the importance of glycosylation, only recently have these complex post-translational modifications been extensively studied, primarily owing to availability of new chemical and MS-based technologies.

This review provides a summary of glycobiology, its incorporation in cancer liquid biopsy for biomarker discovery, and the potential of novel technologies for analyzing the glycoproteome in clinical oncology. The recent breakthroughs in this field carry significant implications for personalized cancer care and enhanced clinical decision-making.

## Enhancing liquid biopsies with glycoproteomic insights

### Liquid biopsies

Liquid biopsy, the analysis of tumor-derived biomarkers in body fluids, offers a promising non-invasive approach to detect cancer and monitor treatment response. To date, most liquid biopsy research has focused on analyzing ctDNA [17, 18], CTCs [17, 19], or exosomes [20] isolated from blood. However, despite great potential, these types of liquid biopsies have shown limited utility in early cancer detection. This limitation mainly stems from technical challenges associated with sensitivity and specificity in light of tumor heterogeneity [21, 22].

Specifically, a key challenge for ctDNA- and CTC-based liquid biopsy is the rarity of these biomarkers, leading to detection sensitivity issues especially in early-stage disease, where ctDNA is present in fewer than 2 copies per mL of plasma [23]. CTCs shed from tumors can be present at concentrations as low as a few cells per mL of blood [24, 25] and isolating intact, viable CTCs from the background of billions of blood cells represents a significant technical hurdle [25, 26]. Additionally, detection of ctDNA of low mutant allele fractions remains a challenge [23]. Beyond rarity, circulating DNA bearing tumor-related signatures can also result from non-malignant processes or normal biological variation, causing decreased specificity for malignancy [21, 23, 27]. To our best knowledge, there are currently no diagnostic tests that address these limitations of DNA-based liquid biopsies, rendering the utility for screening population screening of “average risk” individuals uncertain. More work is needed to understand the (sub)populations that would benefit most from screening, or how liquid biopsies further guide ongoing clinical trials to identify the patient populations likely to respond to therapies.

### Introduction of glycoproteins

**Fig. 1:**
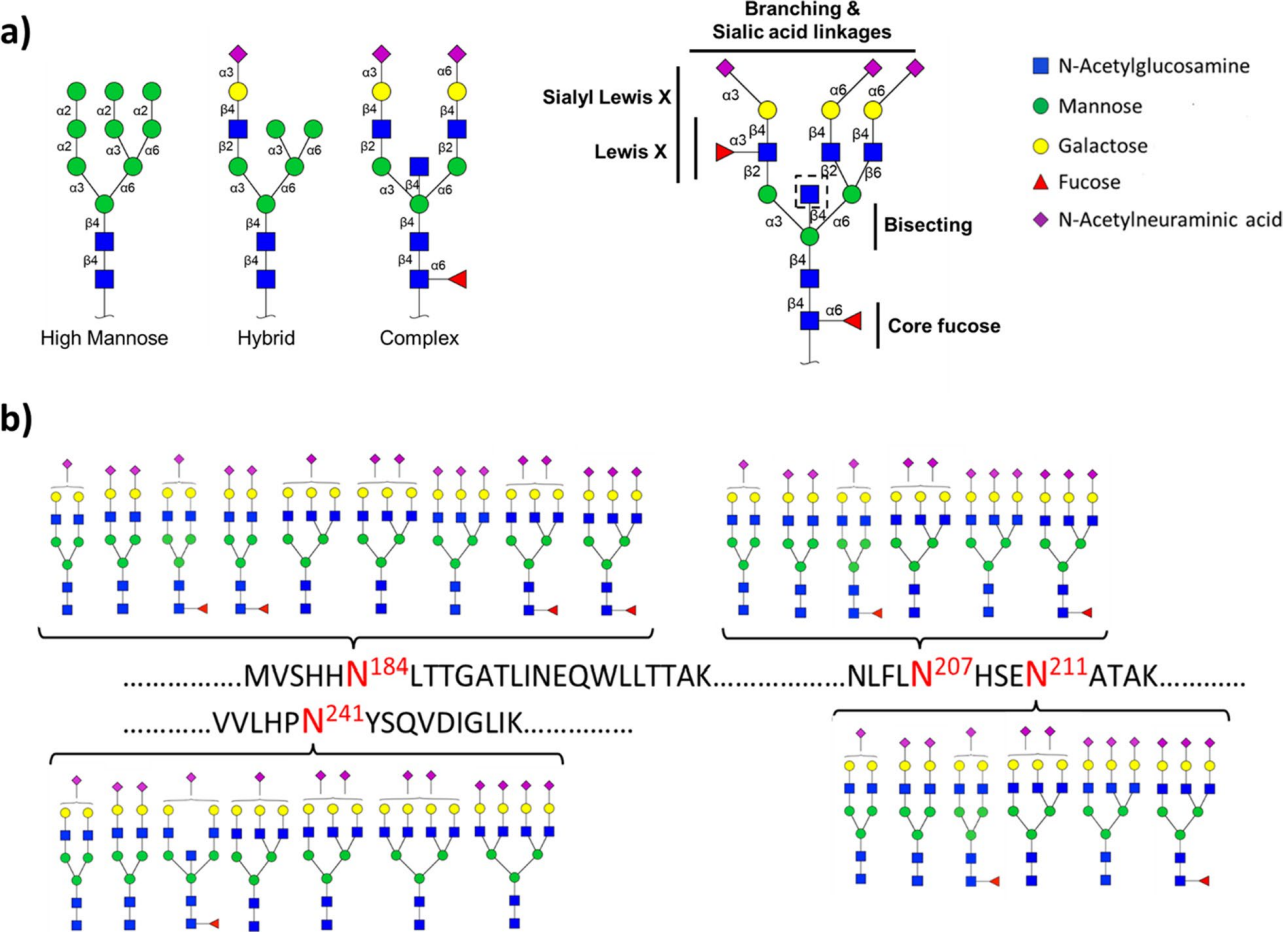
**a** Symbolic representation of N-linked glycan core and representative oligomannose, complex, and hybrid N-linked glycans; **b** Haptoglobin *N*-glycosylations sites. Symbols: blue square – N-acetylglucosamine (GlcNAc); yellow square – N-acetylgalactosamine (GalNAc); green circle – mannose; yellow circle – galactose; red triangle – fucose; purple diamond – N-acetylneuraminic acid (NeuAc, sialic acid)

Enzymatic alterations of a protein after ribosomal synthesis are called post-translational modifications (PTMs) [28]. PTMs can impact structural and functional aspects of proteins, including protein stability, solubility, polarity, and folding [29, 30]. One of the most common and complex PTMs is protein glycosylation: the covalent attachment of glycans to a protein [31]. Most secreted and cell-surface proteins are glycosylated, and in many cases the glycan structures comprise the majority of the glycoprotein’s mass. The glycans on these extracellular proteins can affect the interactions of the protein with other proteins or the extracellular matrix, and they can also engage directly with glycan-binding receptors on other cells or in circulation. In this manner, glycosylation plays a key role both in normal and pathological processes such as cell trafficking, including tumor cell metastasis, and immune cell recognition of cells in the tumor microenvironment [32]. One of the most common forms of glycosylation is *N*-linked glycosylation, which comprises a glycan attached to the side chain nitrogen of an asparagine residue in an Asn-X-Ser or Asn-X-Thr sequon, where X can be any amino acid except proline [33, 34]. *N*-linked glycans share a common core structure on the reducing end consisting of five monosaccharide residues, two N-acetylglucosamines and three mannoses. The core structure is further extended with additional monosaccharides through glycosidic bonds, forming high mannose, complex, or hybrid type structures (Fig. 1a). Analysis of N-glycans is further simplified by the consensus sequence for a glycosylation site. Another form of glycosylation is O-linked glycosylation. In contrast to N-glycans, O-glycans represent a greater challenge for structural analysis as there is no unique consensus sequence for O-glycosylation. The difficulty of O-glycan analysis also lies in the lack of universal enzymes to release O-glycans. There are enzymes that can release mono- and disaccharides from proteins but not larger more complicated structures [35, 36]. Biosynthesis of N-Glycans and O-glycans include a series of competing enzymatic steps through nontemplate driven processes. Table 1 summarizes some key cancer-associated enzymes in the N- and O-glycosylation pathway. Each protein can have multiple glycosylation sites with different glycan structures (or glycoforms) at each site. *N*-linked glycans are attached to proteins co-translationally and concurrently with protein folding. Indeed, *N*-linked glycosylation is a key process that promotes correct folding and trafficking of proteins in the secretory pathway. Beyond their influence on the protein to which they are attached, *N*-linked glycans also contribute to a wide range of biological processes such as intra- and intercellular signaling and interactions with receptors on immune cells. Their central roles in multicellular biology have motivated considerable effort in the past two decades toward the comprehensive study of glycans and glycoproteins [35, 37]. Nonetheless, glycobiology has lagged other branches of molecular biology due to the significant complexity of these biopolymers compared to structurally simpler proteins and nucleic acids, which are linear, more easily sequenced, and amenable to direct genetic manipulation and amplification.

**Table 1:** Key enzymes involved in the cancer-associated N- and O-glycosylation pathway

Enzyme	Description	Clinical Significance	Refs.
Β-galactoside α2,6-sialyltransferase I (ST6Gal-I)	A dds sialic acid to the terminal galactose of *N*-glycosylated proteins. Mediates synthesis of α2,6-sialylated lactosamine (Sia6LacNAc)	Altered expression in various malignancies including colon and ovarian cancer	[42]
Fucosyltransferase VIII (Fuc-TVIII)	Catalyzes the addition of fucose in alpha 1–6 linkage to the innermost GlcNAc residue of an N-linked glycoproteins and mediates core fucosylation	Overexpression of this enzyme is associated with tumor development and progression in lung cancer and breast cancer	[43–45]
Fucosyltransferase VII (Fuc-TVII)	Mediates terminal fucosylation (addition of Fuc in α1,3 linkage to an α2,3-sialylated type 2 chain and giving rise to specific Lewis antigens, such as SLe^x^)	Enhanced expression of SLe^x^ in adult T cell leukemia cells has been shown to be dependent on Fuc-TVII activity	[46]
Fucosyltransferase VI (Fuc-TVI)	Mediates terminal fucosylation (addition of Fuc in α1,3 linkage to an α2,3-sialylated type 2 chain)	Altered expression in breast cancer	[47]
N-acetylglucosamine transferases (β1,3GlcNAc transferase)	I nitiates production of the Lacto series that can carry multiple glycan epitopes such as blood group antigens	Altered expression in colon cancer	[48]
N-acetylglucosaminyltransferase V (GnT-V)	Responsible for branching GlcNAc *N*-glycans	GnT-V-mediated glycosylation regulates the colon cancer stem cell compartment and tumor progression	[49]
N-acetylglucosaminyltransferase III (GnT-III)	Catalyzes the addition of bisecting GlcNAc N-glycans in a β1,4-linkage, suppressing additional elongation of *N*-glycans such as the β1,6-branching structures	Involved in the suppression of lung cancer metastasis	[50]

An example of widely studied glycoproteins are haptoglobins (Hp), a hemoglobin-binding glycoprotein secreted by the liver (Fig. 1b). As a positive acute-phase response protein and the ninth most abundant protein in blood, Hp plays an important role in different biological processes. Hp’s primary physiological function is to bind free hemoglobin and suppress its oxidative activity [38]. As well, Hp has immune modulatory activity and can trigger the angiogenesis pathway. Structurally, Hp is a tetramer consisting of two heavy β-chain and two light chains: α1 and α2. Heavy and light chains of Hp are covalently linked to each other by disulfide bonds. There are four *N*-glycosylation sites on Hp’s 245-amino acid β-chain, positioned at Asn184, Asn207, Asn211 and Asn241. Hp’s α-chains possess no known glycosylation sites [39].

In recent years, many studies have focused on changes in Hp’s *N*-glycosylation pattern that are associated with different diseases, such as inflammatory disorders and malignancies. In cancer, the altered glycosylation of Hp is manifested at different levels such fucosylation (the addition of fucose residues to an underlying glycan), sialylation (the addition of sialic acid residues to an underlying glycan), branching, and the presence of so-called Lewis antigens (glycan structures with fucose attached to an N-acetyllactosamine core) [40]. Branching of *N*-glycans can be augmented by extensions of the core structure with GlcNAc residues through different linkage positions, leading to the production of bi-, tri‐ or tetra‐antennary glycans. Alterations in fucosylation, sialylation, and branching of Hp have been reported in different types of cancers [39]. For example, patients with hepatocellular carcinoma (HCC) display changes in α1-6 fucosylation and α2-6 sialylation of this abundant serum glycoprotein [41].

### Neoplasia-induced hepatic reprogramming

**Fig. 2:**
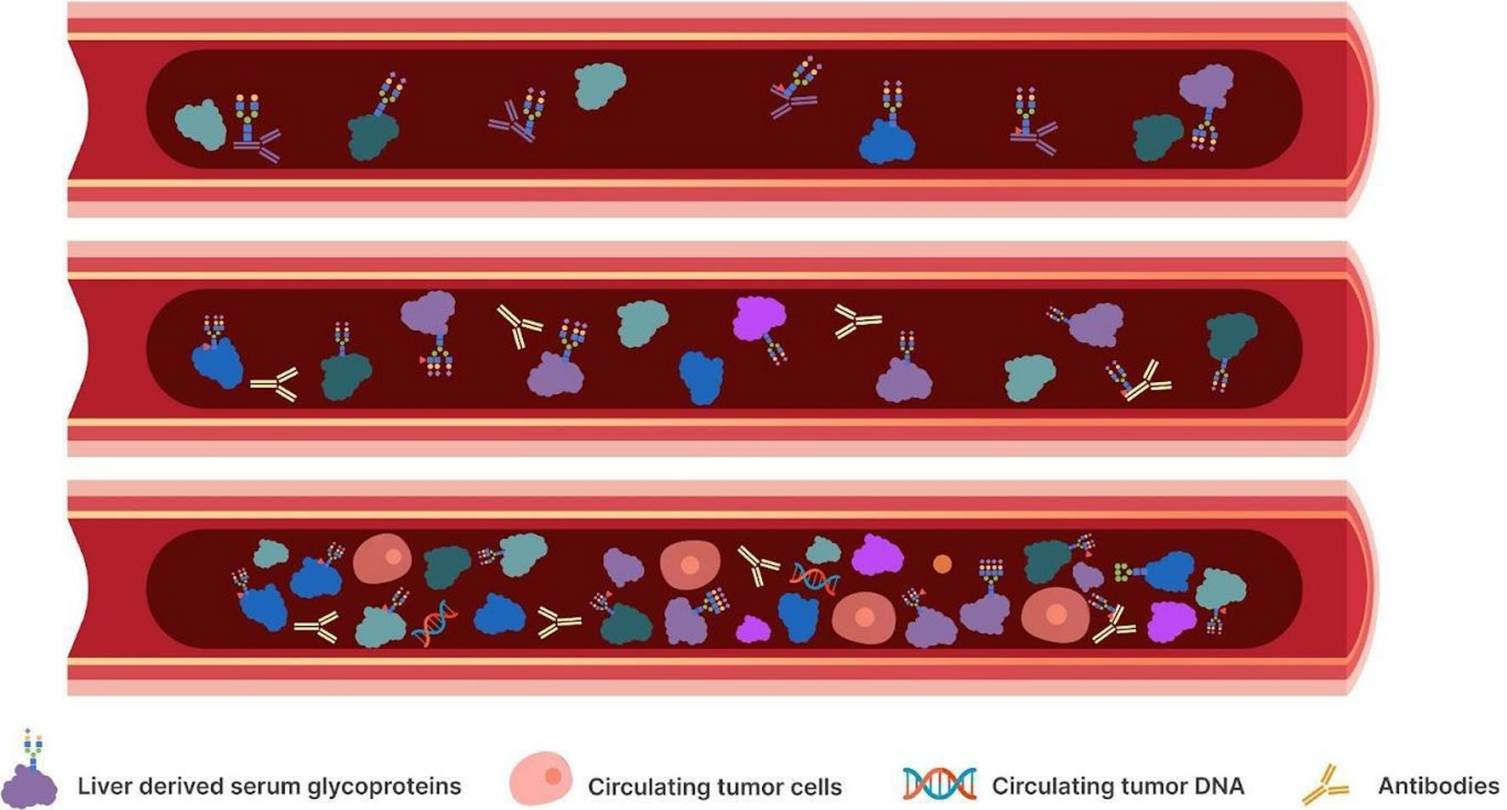
Schematic overview of neoplasia-induced hepatic reprogramming. Top vessel provides a snapshot of homeostatic circulating glycoproteome. Mid vessel schematic depicts early hepatic reprogramming, including changes in glycosylation and abundance of circulating glycoproteins, of circulating liver-derived glycoproteome in pre-cancerous state. Bottom vessel depicts neoplasia-induced hepatic reprogramming with even further increase in glycosylation remodeling and abundance increase. At this stage tumor derived circulating DNA and circulating tumor cells can be detected in circulation

As a sentinel organ filtering blood from the entire body, the liver is strategically positioned to detect, react, and potentially amplify the signals of distant tumors through changes in protein production and secretion. Tumorigenesis and cancer progression in the organs and tissues of human body can significantly impact the profile of hepatocytic synthesis and release of proteins including majority of circulating plasma proteins, leading to a “neoplasia-induced hepatic reprogramming” (Fig. 2) [51–53]. Thus, a comprehensive analysis of this ‘neoplasia-induced hepatic reprogramming’ via the hepatocyte-derived proteome represents a promising liquid biopsy approach to assess host response during oncogenesis and enable early cancer detection. For example, acute phase proteins including C-reactive protein (CRP), serum amyloid A (SAA), haptoglobin, and alpha-1-antitrypsin are dramatically upregulated in response to an inflammatory insult [39]. These hepatocyte-secreted proteins directly enter the bloodstream, serving as systemic biomarkers of a potentially local pathology [54].

Beyond expression changes, aberrant glycosylation of hepatocyte-derived proteins occurs during malignancy [55, 56]. In fact, it is becoming increasingly recognized that the extent and type of glycosylation remodeling of acute phase proteins is unique for different pathologies, as opposed to a more general response as is the case of CRP in inflammatory state [57]. Glycans attached to proteins directly reflect cellular metabolism and homeostasis. For instance, the liver preferentially synthesizes specific glycoforms of alpha-1-acid glycoprotein in certain pathologies [58–61]. Increased branching, sialylation, and fucosylation across the plasma *N*-glycome are linked to tumor presence and progression [62–64].

While any individual protein change, including its glycosylation pattern, is unlikely to demonstrate sufficient specificity to serve as a biomarker on its own, global profiling of the plasma proteome enables high sensitivity detection of physiological states. For instance, mass spectrometry (MS) proteomics generates snapshots of hundreds to thousands of plasma proteins simultaneously and can provide detailed information on their glycosylation status. Coupled with machine learning algorithms, multivariate changes in hepatocyte-derived proteins discern subtle systemic effects of early oncogenesis. For example, large scale serum glycoproteomic profiling revealed unique signatures in clinical cohorts of malignant melanoma patients enabling immune checkpoint therapy response prediction [65]. A similar approach was used to detect advanced colonic adenomas as well as colorectal cancer (CRC) with high specificity and sensitivity [66]. Beyond MS, emerging proximity extension and aptamer assays directly quantify plasma proteins without relying on antibodies [67–69]. These technologies enable rapid, multiplexed measurement of hepatocyte-derived factors.

While detection sensitivity achieved by analyzing liver-derived serum glycoproteins seems high, more rigorous verification and validation through large, prospective cohorts is needed. Perhaps the most pressing question in liquid biopsy of circulating (glyco)proteins is determining specificity for tissue of disease origin, or the ability to detect actual tumor derived circulating (glyco)proteins. Nonetheless, increased awareness of the liver's role as a proxy amplification signal of systemic pathologies represents a promising strategy for liquid biopsy screening. We foresee that further integration of (glyco)proteomics, metabolomics and other complementary omics approaches offer exciting possibilities for unlocking the full potential of plasma-based liquid biopsies.

## Glyco-analytical approaches

The potential of glycoproteins as cancer biomarkers has been recognized for more than four decades. However, outside of a handful of discrete molecules such as PSA and CA-125, their translation into clinical practice has been limited by inherent challenges, including the biological complexity of glycoproteins and limitations of analytical techniques [29]. The comprehensive analysis of glycoproteome requires the identification of the glycan structures as well as the proteins and sites to which they are attached. Over the past few years, multiple glyco-analytical approaches have been undertaken for the characterization of glycans and glycoproteins. Some of these approaches make use of monoclonal antibodies and lectins as highly specific glycan-binding proteins in ELISA formats [70, 71]. These lectin-antibody sandwich assays can be used to distinguish disease-associated from normal glycosylation patterns with a reasonable level of sensitivity and specificity. However, they have limited information content, which does not include the sites on a protein to which glycans are attached. Mass spectrometry (MS) techniques offer a wide range of additional capabilities and are now the key technology in identifying changes in protein glycosylation associated with disease. MS is a robust technique commonly used in cancer biomarker discovery due to its high sensitivity, compatibility with diverse biological matrices, potential for scaling, capability of providing structural information on very small amounts of biological samples, and large instrumental dynamic range. Over the past few years, MS-based methods which provide accurate mass and structural information have contributed significantly to the study of glycans and glycoproteins through glycomics (comprehensive characterization of glycan profile of biological samples) and glycoproteomics (comprehensive profiling of glycopeptides which provides both glycan and protein information) analyses.

### Glycan analysis

Glycomics is the global analysis of glycans that are typically analyzed after cleavage from underlying protein (or lipid) scaffolds using enzymatic or chemical methods [32]. While glycomics are less complicated than glycoproteomics, such analyses provide information regarding aberrant glycosylation changes associated with disease. Mass-spectrometry based glycomics, in conjunction with exoglycosidases and database searches can provide valuable in-depth information about glycans. [72] *N*-Glycans are typically released from glycoproteins using an enzyme, peptide *N*-glycosidase F (PNGase F). PNGase F cleaves the linkage between the core GlcNAc and the asparagine residue in the NXT/S (X ≠ P) sequon [73]. PNGase F works for all classes of *N*-glycans, except the ones with α(1,3)-linked core fucose residues as observed in certain non-mammalian organisms. PNGase A is another enzyme that can be used to release all core fucosylated *N*-glycans with or without α (1,3)-linkage [74].

*N*-Glycan release is usually followed by a separation step to increase the specificity and sensitivity of glycan analysis prior to MS analysis. An efficient separation technique is often needed to characterize the structural heterogeneity of glycans, especially for complicated biological samples. Porous graphitized carbon (PGC), reversed phase (RPLC), and hydrophilic interaction chromatography (HILIC) are among the most common separation techniques that have been applied for glycan analysis [75–78]. Ruhaak et al*.* reviewed the key characteristics of each separation method [35].

Separated glycans are then identified using different MS-based techniques such as electrospray tandem mass spectrometry (ESI–MS/MS) or matrix-assisted laser desorption/ionization time-of-flight tandem mass spectrometry (MALDI-TOF MS/MS). Native mass spectrometry offers high sensitivity and ability to provide glycan structural information [79–81]. For glycan quantitation purposes, multiple reaction monitoring (MRM), which improves detection sensitivity by reducing chemical background noise and results in the identification of low abundance glycans, is among the most reliable MS-based methods [82]. While recent advancement in mass spectrometry-based methods and instrumentation have contributed significantly to the study of glycans, full structural analysis of glycans is still challenging. Different glycan structures might have the same mass and coelute on separation systems. Therefore, manual validation of a structural assignment from one technology using an orthogonal technology is required. Advancement of informatic tools in future can further alleviate this bottleneck.

### From glycans to glycoproteins

A key question that often follows the identification of glycans by glycomics analysis is, which proteins carry the identified glycan structures? Protein glycosylation is known to be protein-specific, which means that different proteins can be glycosylated with different structures modifications even when they expressed in the same cell. Different factors such as common sequence motifs, protein structural conformation, and unique physicochemical patches surrounding the glycosylation site can potentially contribute to the protein-specific nature of glycosylation. However, the exact molecular basis for protein-specific glycosylation is still unknown. Glycoproteomics can provide both glycan and protein information.

### Glycoprotein analysis

Glycoproteomics is based on global profiling of glycopeptides and consists of simultaneous identification of proteins and corresponding glycans [83]. Therefore, glycoproteomics links proteomic and glycomic analyses and ideally enables the identification of the detailed molecular features of all glycoproteins in a biological sample [84]. In such analyses, each glycan- or glycopeptide identified by MS is evaluated independently, or by grouping glycan structures that have similar structural properties into derived glycosylation traits. Most of the current MS-based glycosylation analyses have focused on the large-scale characterization of glycopeptides obtained by proteolytic digestion of complex samples [83]. Glycopeptide analysis by mass spectrometry is challenging due to several factors, for example, glycosylation tends to diminish the intensity of glycopeptide signals compared to signals from unmodified peptides. In addition, different glycoforms of the same peptide can dilute the intensity of the MS signal over several species [85]. Like glycomics analysis, different enrichment strategies are applied in glycoproteomics workflows to reduce competition for charge with the highly abundant and easily ionizable non-glycosylated peptides. Among common enrichment methods, multi-lectin affinity and HILIC are commonly used for untargeted glycoproteomics. On the other hand, immunoaffinity and single-lectin affinity are mostly applied to the targeted enrichment of one or a small group of glycoproteins [86]. Over the past few years, several groups have used various enrichment techniques to overcome some of the above-described issues associated with the analysis of glycoproteins [87–89]. Enriched glycopeptides are commonly separated using reversed-phase liquid chromatography (RPLC) prior to introduction to MS. RPLC separates glycopeptides based on the interaction of the stationary phase with the peptide backbone and not the glycan part. Therefore, it cannot separate different glycoforms of the same peptide backbone. Moreover, highly hydrophilic glycopeptides show poor retention on RPLC [90]. To address these limitations of RPLC, alternative separation methods such as capillary electrophoresis (CE) and ion mobility (IM) have been applied to separate glycopeptides [91, 92]. Untargeted discovery of cancer-associated glycopeptide biomarkers requires high resolution MS instrumentation, such as an orbitrap MS instrument, using data-dependent or data-independent acquisition (DDA or DIA) methods [93, 94]. Currently the best suited MS approach for the analysis of targeted cancer-related glycopeptides is multiple reaction monitoring (MRM). During MRM, predetermined precursor glycopeptides are selected for further collision-induced dissociation and the appearance of several diagnostic product ions are monitored. Therefore, MRM offers high analytical specificity and sensitivity. MRM analysis with addition of isotopically labeled internal standards enables absolute quantification of target glycopeptides [74, 87, 95].

## Biomarker development

While with recent advancement of analytical technologies, there has been considerable improvements in understanding the role of glycosylation in cancer, there is still a large gap between our findings of glycoprotein biomarkers, often with diagnostic performance, and full clinical implementation of the cancer biomarkers. Development of biomarkers involves multiple steps. First, biomarkers should be discovered and validated using small sample sizes. Candidate biomarkers are then subjected to two types of validation following the discovery phase: 1) Analytical validation, which evaluates how accurately and reproducibly the analyte(s) of interest is measured within the patient samples; 2) Clinical validation, which aims to assess the robustness of the test result and its correlation with the clinical phenotype [96]. Clinical validation phase typically requires a relatively large and independent sample set to demonstrate the clinical validity and utility of the biomarker. Key considerations in biomarker discovery and validation study design include the patient population, prevalence of the disease, sample source (retro- vs. prospective), sample size, and sample type.

### Tumor-originated protein biomarkers

Conventionally, cancer diagnostic tests are based on the levels of single biomarkers. Although most of the FDA-approved protein biomarkers for cancer are monitored based on their concentrations at the protein level only, the majority of these proteins are found or predicted to be glycosylated and their specific glycosylated forms associated with cancer progression have shown higher performance than protein levels alone in either clinical settings or research laboratories [74, 97]. The most common glycoprotein biomarkers and their cancer-associated glycoforms are listed in Table 2. Glyco-variant based assays for these protein biomarkers and their diagnostic power compared to conventional tests based on protein concentrations have been reviewed recently [29]. Analytical approaches for these glycosylated single protein biomarkers usually involve using an antibody to capture the protein, followed by glyco-profiling using lectin array to discover cancer-specific glycoforms, and then a selected lectin to detect the one specific group of glycoproteoforms associated with cancer [70, 98, 99]. For example, alpha-fetoprotein (AFP) is a well-established protein biomarker for hepatocellular carcinoma (HCC), and its core-fucosylated form AFP-L3 has been approved by FDA as a biomarker widely used in combination with the total AFP concentration for risk assessment of patients with chronic liver disease for development of HCC [100–102]. In a study of 689 patients with cirrhosis and/or chronic hepatitis B including 44 diagnosed HCC patients, the area under the receiver operating characteristic (ROC) curve (AUC) by combining AFP and AFP-L3 is 0.83 compared to 0.77 with AFP alone [103].

**Table 2:** Representative glycoproteins as cancer biomarkers

Marker	Full name	Cancer type	Cancer-associated glycoforms*		Refs.
AFP	α-Fetoprotein	Liver	Core-fucosylation (AFP-L3)		[102, 104, 105]
β-hCG	β-Human chorionic gonadotropin	Testicular, ovarian	Tri-antennary branching		[106, 107]
MUC1 (CA15-3 /CA27-29)	Mucin1 (Cancer antigen 15–3/27–29)	Breast	Sialylation & High-mannose		[108–110]
CA19-9	Carbohydrate antigen 19–9 or cancer antigen 19–9	Pancreatic, ovarian, gastrointestinal	Sialyl Lewis-a		[111–113]
MUC16 (CA125)	Mucin16 (Cancer antigen 125)	Ovarian	Sialyl-Tn antigen		[114–116]
CEA and other CEACAMs	Carcinoembryonic antigen-related cell adhesion molecules	Colorectal, pancreatic, lung	Fucosylation & Bisection and branching		[117–121]

^*^R – glycan chain; Ser/Thr – serine or threonine; blue square – N-acetylglucosamine (GlcNAc); yellow square – N-acetylgalactosamine (GalNAc); green circle – mannose; yellow circle – galactose; red triangle – fucose; purple diamond – N-acetylneuraminic acid (NeuAc, sialic acid)

Another tumor-originated glycoprotein that has been extensively studied is prostate-specific antigen (PSA). PSA has remained the gold standard biomarker for prostate cancer screening although PSA testing has been controversial due to its poor performance [122, 123]. PSA has a single *N*-glycosylation site occupied by complex-type glycans. Detection of specific glycoforms such as ɑ2-3 sialylated, core-fucosylated, or LacdiNAc-modified glycans has shown potential as a novel tool for improving the clinical utility of PSA test [124–128]. Diagnostic performances of these glycoforms compared with total PSA alone are summarized in Table 3. In two small-scale studies with 100 or fewer subjects, ɑ2-3 sialylated PSA had much higher AUCs of 0.834 and 0.971 compared with the AUCs of total PSA at 0.506 and 0.806 respectively [129, 130]. In another relatively large-scale study, a total of 414 patient samples with 100 samples in the training set and 314 samples in the test set were analyzed using a magnetic bead-based immunoassay. With a sensitivity of 90.6%, the test set specificities were at 20.5% for total PSA and 64.2% for ɑ2-3 sialylated PSA [131]. Similar trends of increased performance were observed for core-fucosylated PSA and LacdiNAC-glycosylated PSA where AUCs are improved to 0.94 and 0.851, respectively [132, 133]. In more recent larger cohort studies, the trends of performance improvements are validated although not as significant. With a sensitivity of 90%, the specificity of core-fucosylated PSA was 36% for a cohort of 252 men, and that of LacdiNAc-glycosylated PSA was 48.6% for a cohort of 718 men [134, 135]. Findings from these independent studies have demonstrated the potential of glyco-PSA for prostate cancer detection. On the other hand, PSA has also been comprehensively studied by MS methods owing to its smaller protein size and simplicity of glycosylation profile with only one glycosylation site. In 2012, an interlaboratory study was conducted to compare MS-based analytical methods for the characterization of human seminal PSA and PSA-high isoelectric point isoform [136]. The collected datasets demonstrated the high heterogeneity of PSA glycosylation with 61 glycoforms observed by multiple laboratories. However, validation on large prospective cohorts is needed to demonstrate the clinical utility of these novel glycoforms as biomarkers.

**Table 3:** Specific glycoforms of PSA improve its diagnostic performance compared to total PSA

Glycoform*	Assay performance		Sample size		Refs.
	Total PSA	Specific glycoform of PSA	Controls	Cases	
ɑ2-3 Sialylation	AUC: 0.506 Sensitivity: N/A Specificity: N/A	AUC: 0.834 Sensitivity: 80.0% Specificity: 72.0%	50	50	[130]
	AUC: 0.806 Sensitivity: N/A Specificity: N/A	AUC: 0.971 Sensitivity: 85.7% Specificity: 95.3%	29	50	[129]
	(Test set) AUC: 0.60 Sensitivity: 90.6% Specificity: 20.5%	(Test set) AUC: 0.84 Sensitivity: 90.6% Specificity: 64.2%	Training: 50 Test: 176	Training: 50 Test: 138	[131]
Core-fucosylation	AUC: 0.89 Sensitivity: N/A Specificity: N/A	AUC: 0.94 Sensitivity: 90% Specificity: 95%	29	44	[133]
	AUC: 0.629 Sensitivity: N/A Specificity: N/A	AUC: 0.729 Sensitivity: 90% Specificity: 36%	87	165	[134]
LacdiNAc	AUC: 0.712 Sensitivity: 90.0% Specificity: 27.0%	AUC: 0.827 Sensitivity: 90.0% Specificity: 48.6%	Prostate biopsy cohort: 347	Prostate biopsy cohort: 371	[135]
	AUC: 0.559 Sensitivity: N/A Specificity: N/A	AUC: 0.851 Sensitivity: 88.4% Specificity: 40.7%	27	44	[135]

*Symbols: blue square – N-acetylglucosamine (GlcNAc); yellow square – N-acetylgalactosamine (GalNAc); green circle – mannose; yellow circle – galactose; red triangle – fucose; purple diamond – N-acetylneuraminic acid (NeuAc, sialic acid)

Human carcinoembryonic antigen (CEA) is the most frequently used biomarker for colorectal cancer screening and monitoring. Unlike PSA, CEA has 28 potential *N*-glycosylation sites, making it an even more heterogeneous and variable target to characterize. Early studies revealed tumor-specific glycosylation of CEA and their interaction with dendritic cells indicating the potential of monitoring these glycoforms as novel biomarkers for early detection of CRC [137, 138]. Lectin array analysis of colorectal cancer samples showed the levels of fucosylated and mannosylated glycans are increased in tumor-associated CEA while branched and bisecting *N*-glycans are decreased [117]. The site-specific glycopeptide analysis of CEA remains challenging, but emerging LC–MS techniques have made exploratory studies possible. In one study, CEA proteins purified from human colon carcinoma and human liver metastases of colorectal carcinoma cells were characterized with 893 different *N*-glycopeptides and 128 unique *N*-glycan compositions identified from 21 out of 28 potential *N*-glycosylation sites. The site-specific glycosylation changes such as increased bisection and branching, incomplete galactosylation or LacNAc elongation on highly branched structures, moderate levels of sialylation, and extremely high levels of fucosylation were observed, providing another layer of potential in differentiating tumor-specific CEA [118]. Nonetheless, similar comprehensive profiles of serum CEA are still unavailable due to the low concentration of circulating CEA in blood. Besides CEA, several other glycoproteins of the carcinoembryonic antigen-related cell adhesion molecule (CEACAM) family are emerging as cancer biomarkers, such as CEACAM1, CEACAM5 (synonym of CEA) and CEACAM6 for pancreatic cancer [119, 120].

Cancer antigen 125 (CA125) for ovarian cancer as well as cancer antigen 15–3 (CA15-3) and cancer antigen 27–29 (CA27-29) for breast cancer are based on mucin proteins mucin-16 (MUC16) and mucin-1 (MUC1), respectively [139]. These mucin proteins are heavily glycosylated, and lectin-based studies have shown the associations of cancer progression with altered mucin glycosylation (Table 2). However, few studies have been conducted to reveal their site-specific molecular structures due to the challenges to process them for mass spectrometry analysis [140]. Comprehensive structural analyses have only been made possible in the past couple of years through the characterization and commercialization of mucinases such as StcE [141, 142]. The rise of mucinomics enabled by advancing sample preparation and MS techniques, although at its early stage, has provided the potential to unravel the complex relationship between mucins and cancer progression.

Despite the potential of glycosylation analysis of tumor-associated single protein biomarkers, the clinical applicability of these novel biomarkers remains challenging and the reported study scales have been small. Lectin assays have high sensitivity to detect overall glycosylation changes with high throughput. Nonetheless, the specificities of lectins are often broad and vaguely defined, which limits the improvement in assay performance when specific glycoforms are identified as more potent biomarkers [71, 143]. MS-based methodologies provide higher specificity that have made comprehensive site-specific profiling of these glycosylated protein biomarkers possible, but technical barriers towards clinical utility include the need of large sample volume to purify sufficient proteins, non-specific binding of interferences to antibodies during purification due to the low concentrations of these target proteins in serum, and the relatively high cost and low throughput compared to lectin assays. Therefore, to achieve breakthroughs in diagnostic performance, development of novel biomarkers with clinical applicability are necessary.

### Glycoproteomics-based multi-marker assays

While single biomarkers can provide easily interpretable readouts to physicians and patients, multi-marker tests can often achieve higher diagnostic power [144, 145]. Recent advances in MS-based technologies have provided an opportunity to discover and validate a panel of glycoproteomic biomarkers for disease detection and monitoring. In most settings, biomarker discovery is performed on retrospective study samples while specimens and data collected from prospective trials are required for clinical validation. Therefore, new glycoproteomics-based multi-marker assays must prove their analytical applicability and validity for large and independent patient cohorts [146, 147].

**Fig. 3:**
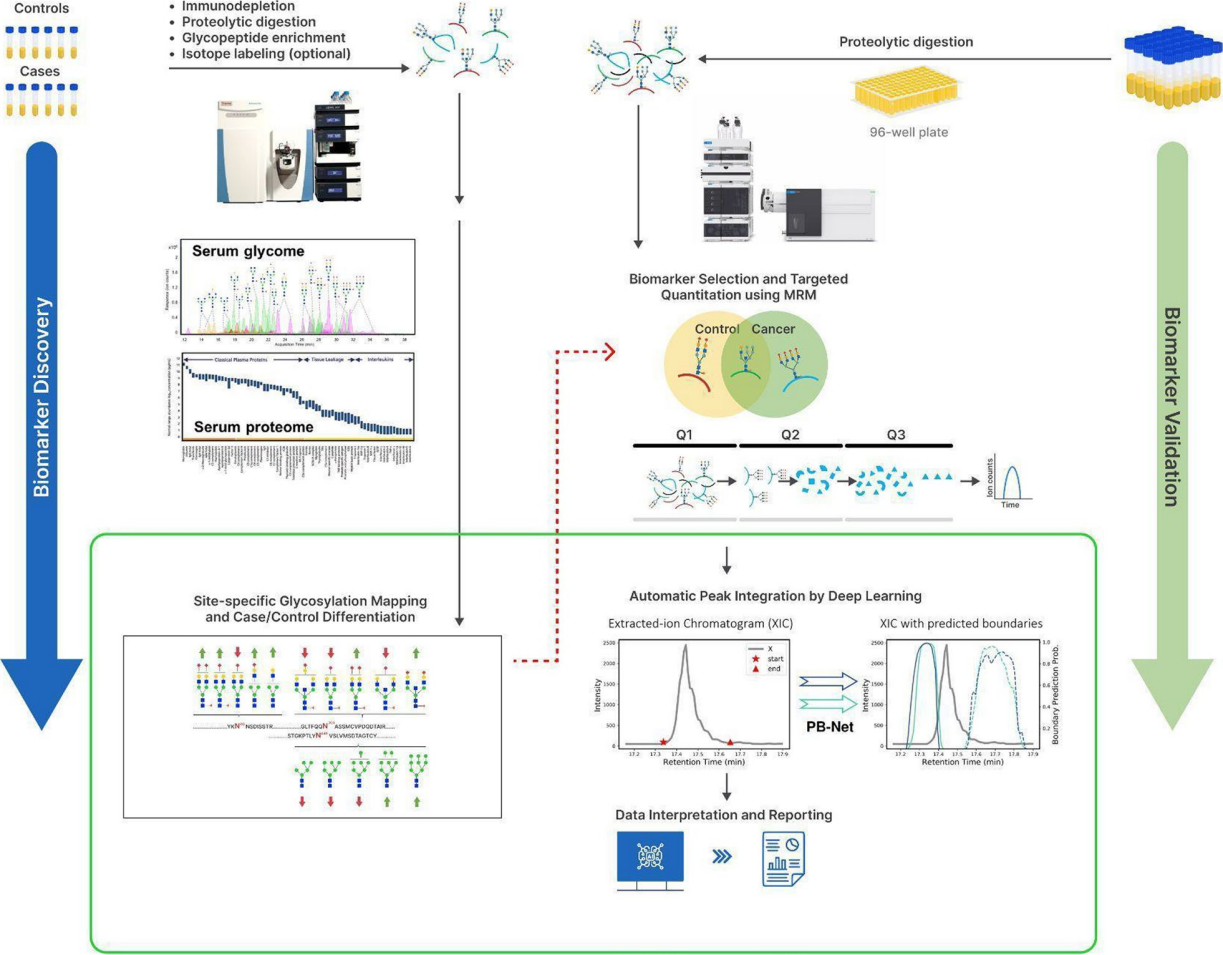
Multi-marker assay development through biomarker discovery and validation workflows enabled by LC–MS and AI technologies

Analytical approaches for glycopeptide quantification have been extensively reviewed previously [148, 149]. In general, a small set of cases and controls are studied for biomarker discovery where the serum or plasma samples undergo multiple processing steps such as immunodepletion, proteolytic digestion, enrichment, and optionally isotope labeling and fractionation to achieve glycopeptide-rich samples. Then high-resolution MS instruments are used to identify and quantify as many glycopeptides as possible before glycopeptides with significant differences between the cases and controls are selected as biomarker candidates (Fig. 3). These glycopeptides are then quantified in a targeted manner across large patient cohorts for validation. Among different targeted approaches available, multiple reaction monitoring (MRM) is the gold standard MS method as it provides the highest sensitivity, high throughput, and simplicity of sample preparation and data interpretation.

MRM methods for glycopeptides were initially developed for a handful of protein targets such as immunoglobulins and apolipoproteins [82, 87, 150, 151]. These methods were applied to various cancer cohorts. For example, truncated and galactosylated glycoforms from serum immunoglobulins were found to be associated with ovarian cancer and gastric cancer [152, 153], while fucosylated glycopeptides of haptoglobin and hemopexin were found to be elevated in HCC patients [154]. In 2019, we fully explored the potential of MRM for serum glycopeptide quantitation by expanding the method to quantify over 600 glycopeptides from 50 serum glycoproteins, and later, the method was further expanded to study the glycosylation of over 70 serum glycoproteins simultaneously [155, 156]. This workflow for targeted serum/plasma glycoproteomic analysis is illustrated in Fig. 3 in parallel with the biomarker discovery process. As the first step, serum samples from retrospective or prospective cohorts are transferred into 96-well plates for high-throughput preparation. To reduce variation introduced by sample pre-processing, the samples are handled only by a simple proteolytic digestion procedure and directly injected into a LC coupled with a triple quadrupole mass spectrometer (QQQ-MS) where the peptides and glycopeptides of interest are sequentially isolated, fragmented, and detected as chromatographic peaks. Due to the high heterogeneity of glycosylation and wide dynamic range of glycoforms, conventional peak integration software tools for proteomics are not readily applicable to glycopeptide MRM data. In 2020, an AI-based peak integration platform, PB-Net, was developed by training on a large dataset of over 170,000 expert annotated MRM peaks spanning a wide dynamic range, including both peptides and intact glycopeptides [157]. The peak areas with comparable accuracy as human integration are then exported for each analyte from all patient samples and used to build machine-learning models for cancer diagnosis. This MRM-based workflow has demonstrated its clinical applicability through several use cases including discovering novel glycoproteomic biomarkers for detecting NASH and HCC, differentiating symptomatic and asymptomatic COVID-19, as well as predicting clinical benefit from immune checkpoint inhibitor therapy for metastatic melanoma patients [65, 156, 158]. (Glycopeptides from a group of liver-derived glycoproteins, including α-2-macroglobulin, α-1-acid glycoprotein 1, haptoglobin, α-1-antitrypsin, and complement factor H were found to have pronounced unidirectional quantitative differences among controls, NASH, and HCC [156]. Besides these small scale pilot studies, this workflow has been applied to prospective clinical trials and the development of a laboratory-developed test (LDT) the reproducible measurements of intact glycopeptides by MRM, reliable data processing by AI-based platform, and novel machine-learning algorithms for data interpretation have started a new era for biomarker validation and cancer detection.

## Glycopeptide biomarkers in clinical oncology

Given the improvement in glycoproteomic detecting technology, as well as machine learning and AI pipelines, glycopeptide biomarkers discovery and development emerge as very promising and clinically relevant field in clinical oncology. Broadly, these biomarkers play a critical role in clinical medicine by providing valuable insights into the various disease status and can be categorized into three distinct types: diagnostic, predictive, and prognostic biomarkers. Each type serves a specific purpose and offers unique insights into the management and treatment of cancer and other diseases.

### Examples of diagnostic/screening glycopeptide markers in cancers

Cancer *diagnostic/screening biomarkers* hold the key to early disease detection and accurate diagnosis. Their primary function is to determine the presence of a disease in a noninvasive manner. By analyzing these biomarkers in blood, tissue, or other bodily fluids, clinicians can initiate timely interventions and treatments, greatly enhancing the patient's chances of improved outcomes. Also, the noninvasive nature of diagnostic biomarkers minimizes patient discomfort and allows for swift initiation of appropriate medical strategies. In the next section, we will give some examples of glycopeptide biomarkers and their clinical applicability.

#### Colorectal cancer

**Fig. 4:**
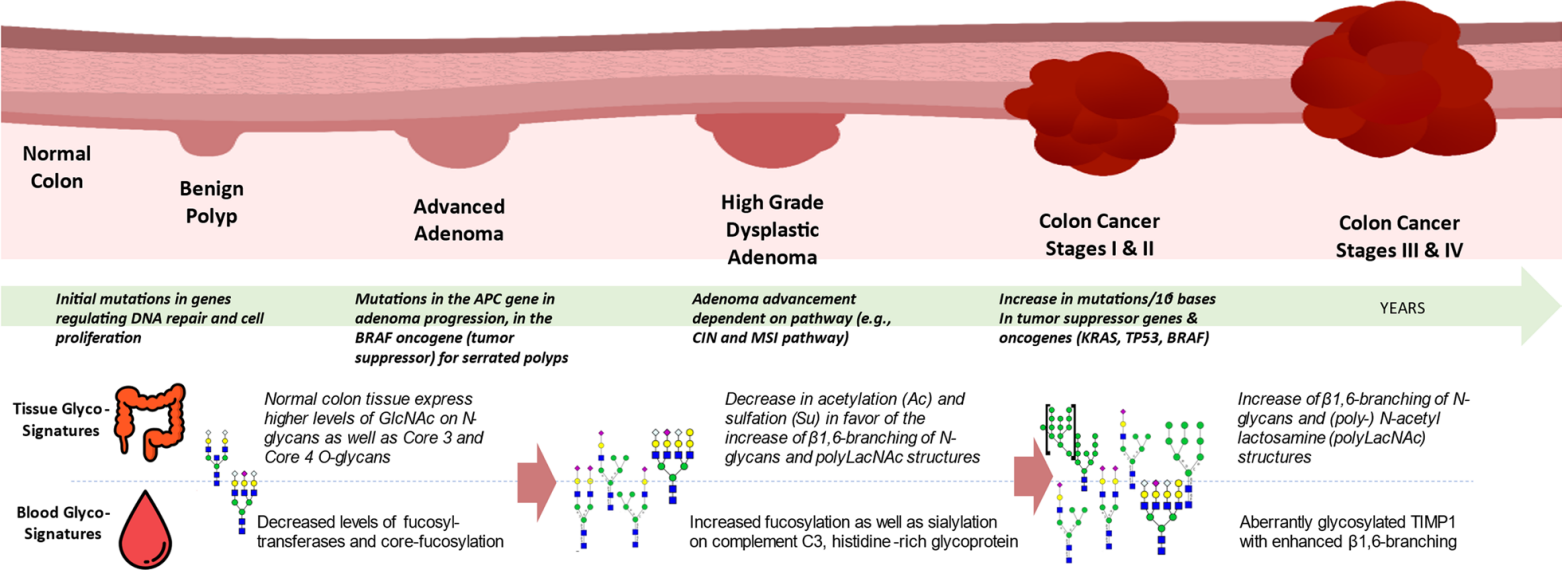
Aberrant glycosylation impacts the host response during the colorectal adenoma-to-carcinoma transformation, affecting cell adhesion, proliferation, signaling, and immune responses to neoplasia. These changes can be measured both on tissue level as in serum [66, 160]

Despite its preventability by proper screening, CRC remains a major cause of cancer-related deaths [159]. Novel, more accurate, less invasive, and cost-effective screening methods are urgently needed. Recent research has highlighted specific biological changes during the transition from adenoma to carcinoma, particularly specific immune responses in the colonic crypt [160]. Abnormal protein glycosylation is a key player in driving these responses, evident in both colonic tissue and circulating glycoproteins (Fig. 4) [160].

During the adenoma to carcinoma sequence, glycosylation patterns within the colonic crypts undergo dynamic shifts (Fig. 4). This influence extends to circulating glycosylated acute-phase proteins and plasma-derived antibodies. Studies focusing on the *N*-glycome in CRC patients have illuminated a connection with branched and poly-LacNAc elongated *N*-glycans, which normalize after treatment [63]. Further investigations into *N*-glycan profiling reveal heightened fucosylation and sialylation on specific proteins, potentially aiding in the detection of CRC [161]. Moreover, disparities in IgG N glycome profiles have been observed between individuals with CRC and those without, offering potential diagnostic insights [162]. Intriguingly, matrix-assisted laser desorption/ionization-MS analysis of patient sera indicates correlations between multi-antennary, sialylated *N*-glycans and cancer progression, while biantennary core-fucosylated *N*-glycans show negative correlations [163]. This not only enhances the accuracy of CRC classification but also suggests possibilities for distinguishing advanced adenomas. Furthermore, glycan-binding proteins such as galectins, including galectin-2, -3, -4, and -8, have been identified at elevated levels in the serum of CRC patients, with galectin-2 demonstrating a positive association with high CRC mortality [164, 165].

The discussed advanced MS technologies and AI-driven data processing have enabled the study of the highly complex field of glycosylation, offering new and scalable possibilities for identifying circulating CRC biomarkers [157]. In a recent case–control study encompassing CRC, AA, and control serum samples, we used LC–MS glycoproteomic quantification and machine learning models with recurrent neural networks to facilitated chromatogram peak integration and molecular abundance quantification [66]. Through this approach, researchers identified 399 statistically significant differentially abundant glycopeptides/peptides when comparing CRC and advanced adenoma samples against healthy subjects. Subsequently, a multivariable classifier model, developed using a subset of six biomarkers, demonstrated strong performance in distinguishing CRC/advanced adenomas from healthy samples. The classifier yielded an Area under the Receiver Operating Characteristic curve (AuROC) of 0.96, reflecting its diagnostic accuracy. This model exhibited high sensitivity for detecting CRC across all stages (89.8%), including stages 1 to 4, as well as for advanced adenomas with and without high-grade dysplasia (90.9%). Additionally, it displayed promising specificity (89%) for normal findings [66].

This innovative glycoprotein-based strategy, empowered by artificial intelligence, showcases compelling clinical efficacy in detecting advanced adenomas and CRC. It offers unique insights into the early host response to polyp formation and immune escape, distinct from current liquid biopsy methods that require significant tumor materials. Prospective studies are underway to evaluate these glycoproteomic markers for AA/CRC detection (NCT05445570).

#### Lung cancer

Lung cancer is a leading cause of cancer-related deaths worldwide which is partly due to a frequent lack of symptoms and signs as well as reliable methods to screen for early-stage disease [166]. Lung cancers are often diagnosed at locally advanced or metastatic stage, the latter of which carries a five-year survival rate below 10% and more than 50% probability of mortality within a year after diagnosis. Current modality of screening involves low-dose computed tomography, but it is limited by the high-false positive rate, exposure to radiation and financial cost [167].

A few studies have investigated the role of glycoproteomic signatures in diagnosis of lung cancer. In one study, targeted glycoproteomic screening using hydrazide chemistry-based capture and enrichment showed varying abundance of 38 glycopeptides from 22 different proteins in patients with non-small cell lung cancer (NSCLC) versus matched controls [168]. A separate study used multiple-lectin affinity chromatography to identify differential levels of 38 serum glycoproteins in the sera of patients of lung adenocarcinoma [169]. In a study using hydrophilic interaction liquid chromatography (HILIC) and weak anion exchange high performance liquid chromatography (HPLC), significant differences in *N*-glycome were observed in patients with lung cancers of any histological types, with most significant alterations seen with di-sialylated and tri- and tetra-antennary glycans [170]. While the area under the curve (AUC) ranged from 0.640 to 0.811 for individual glycan peaks, it performed best with an AUC of 0.938 (85% sensitivity and 86% specificity) when all glycan data were considered. Similar observation was made with increased levels of fucosylated tri- and tetra-antennary structures using an alternative analytical methodology [171]. Notably, some changes were seen as early as in Stage I disease. In a separate study, Fang and colleagues investigated tumor specific *N*-glycosylation sites in Stage I lung adenocarcinoma using tandem mass tag labeling and liquid chromatography with tandem mass spectrometry. A total of 39 differential *N*-glycosylation sites were identified with implications in various biological pathways including cell migration, metabolism and immunity [172]. ITGB3-680 showed the highest diagnostic value with AUC of 99.2% and 95% of both sensitivity and specificity in comparison to the healthy control. Combination analysis of all *N*-glycosylation sites by machine learning model revealed 100% AUC in both training and testing groups. Similarly in small cell lung cancer, proteomic screening revealed significant alterations in fucosylated glycan patterns like that of PON1 with AUC of 0.91 [173]. Recently, similar glycoproteomic platform as used in CRC in the above session that combined mass spectrometry with a proprietary artificial-intelligence-based data processing engine that allows highly scalable interrogation of the glycoproteome in lung cancer was utilized to generate a glycoproteomic classifier that showed high sensitivity and specificity in separate lung cancer and normal tissues [174].

#### Ovarian cancer

Ovarian cancer ranks as the second most common gynecologic malignancy and claims the highest toll among gynecological cancers ranking 4th in leading causes of cancer-related death in US women [175]. While early-stage cancer can be treated effectively with surgery and (neo)adjuvant therapies, its diagnosis is hindered by subtle and nonspecific symptoms like pelvic pain, urinary changes, and abdominal bloating. Such symptoms intensify as the disease advances. Consequently, only 15–20% of cases are caught early, where the survival rate exceeds 90%, while most diagnoses occur at a later stage, yielding survival rates between 17 and 39% [176].

Circulating biomarkers, often involving previously discussed glycoproteins, are combined with other tests for diagnosing ovarian cancer. Elevated CA-125 levels, a glycosylated protein present in blood, can signal ovarian cancer, however, CA-125's performance as a diagnostic biomarker is constrained by its low sensitivity and specificity [177]. Notably, serum CA-125 remains unaltered in 21% of ovarian carcinomas, while elevated levels appear in various other conditions like endometriosis, uterine fibroids, menstruation, or pregnancy. Thus, CA-125 is more suited for monitoring cancer's progress and treatment response [178]. While tests combining CA-125 with other parameters have been developed, they are hindered by complexity and subpar performance. Hence, a critical need remains for highly sensitive and specific noninvasive diagnostic tools for ovarian cancer.

As previously discussed, a significant portion of existing cancer biomarkers are glycosylated proteins, with hyper-sialylation being a consistent glycan alteration observed in cancer. Hyper-sialylation involves the addition of sialic acid to the terminal end of glycoproteins by sialyltransferases. Of the over 20 human sialyltransferases identified, heightened expression of Golgi β-Galactoside α-2,6-Sialyltransferase 1 (ST6Gal1) appears to be linked to various cancers, including ovarian cancer [179–181]. ST6GAL1 modulates intracellular signaling to regulate tumor cell phenotype, in particular, ST6Gal-I upregulation in ovarian cancer cells seems to confer a cancer stem-like cell (CSC) phenotype [181]. Primary ovarian cancer cells from patient ascites or solid tumors sorted for α2-6 sialylation grew as spheroids, while cells lacking α2-6 sialylation remained as single cells and lost viability. In addition, sialylation of EGFR by ST6GAL1 induces receptor activation and modulates trafficking dynamics highly implicated in neoplastic mechanisms [179]. A mechanism for upregulated ST6Gal-I expression in ovarian cancer has recently been suggested whereby SOX2 and ST6GAL1 are coordinately amplified in cancer cells, with the Sox2 protein then binding the ST6GAL1 promoter to further augment ST6Gal-I expression [180].

Multiple glycoproteomic studies have been conducted in ovarian cancer [182–184]. In a recent study, a novel blood-based glycoproteomic approach combining mass spectrometry with machine learning was used to evaluate a glycopeptide classifier for the diagnosis of ovarian cancer [185]. Specific glycopeptide biomarkers were identified that effectively distinguish between individuals with benign pelvic masses and those with malignant ovarian cancer. The classifier exhibited impressive sensitivity and specificity rates of 83.5% and 90.1% in the training dataset and 86.7% and 86.7% in the testing dataset, respectively. Furthermore, it was reported that ovarian cancer patients had elevated levels of fucosylated markers, primarily originating from the liver. Individuals with advanced disease stages (FIGO stage III and IV) showed significantly higher levels of tri- and tetra-antennary glycopeptide markers containing fucose.

These data are encouraging and provide insights into the underlying mechanisms connecting ovarian cancer to the circulating glycoproteome. These results have the potential to guide the development of robust clinical tests for diagnosing and staging of ovarian cancer patients.

### Selected examples of predictive biomarkers

Predictive biomarkers are instrumental in personalized medicine, tailoring treatment plans to the individual patient's characteristics. These biomarkers help determine how a patient is likely to respond to a particular therapy. A 'Companion Diagnostic or CDx' is a test for a predictive biomarker that classifies patients (e.g., tumors) into responders and non-responders, for a specified therapeutic agent. Companion diagnostics are designated as Class III medical devices by the FDA, because the test result equates directly to administration of a drug.

## Predictive to immunotherapy

A limited number of studies have investigated the prognostic utility of glycoproteomic signatures in response to immune checkpoint inhibitors (ICI), which have revolutionized the treatment of various malignancies. A recent study explored predictive ICI biomarkers in melanoma using an advanced platform combining liquid chromatography/mass spectrometry and AI [65]. Glycoproteins were examined in pre-treatment plasma samples from metastatic melanoma patients undergoing different ICI therapies including first or second-line anti-PD-1 monotherapy (pembrolizumab or nivolumab) or anti-PD-1/anti-CTLA-4 combination therapy (nivolumab/ipilimumab). Biomarkers were identified for survival analysis, and patients were categorized by treatment response. Classifiers were developed using a discovery cohort, validated, and externally confirmed. In total, 143 glycoproteomic biomarkers distinguishing early treatment failure from sustained control were found. The subsequent classifier yielded a hazard ratio of 2.7 (p = 0.026) in the training set and 5.6 (p = 0.027) in an independent test set [65]. These markers demonstrated differential expression in patients with short overall survival as compared to those with favorable overall survival outcomes and therefore were used to generate classifiers that identified metastatic melanoma patients. In addition, a specific fucosylation signature in plasma *N*-glycoproteins of patients that do not achieve a durable response to ICI therapy was discovered.

Similarly, several abstracts have demonstrated highly encouraging preliminary findings in advanced NSCLC [186–188]. By utilizing a similar advanced glycoproteomics platform, the most recent study investigated 532 glycopeptide and peptide signatures representing 75 serum proteins in 123 individuals with unresectable stage 3 or 4 NSCLC prior to initiation of standard of care with pembrolizumab monotherapy or combination of pembrolizumab and chemotherapy. Multivariable model-based classifier consisting of 7 glycopeptides and other non-glycosylated peptide biomarkers was able to identify patients who are likely to benefit from ICI with > 95% sensitivity and 33% specificity. This classifier yielded a statistically significant hazard ratio (HR) of 3.6 for predicted benefit with median overall survival (OS) of 13.9 versus 4.2 months in the entire cohort based on its score above and below the cutoff. It similarly performed well with HR of 3.5 and median OS of 13.5 versus 4.5 months in the first-line treated patients. Altogether, these studies confirm the potential of the development of diagnostic tests to guide treatment decisions.

It is worth noting that programmed death-ligand 1 (PD-L1) is often found with significant N-linked glycan moieties in various primary cancers [189]. An important clinical implication of PD-L1 N-linked glycosylation is that it can hinder diagnostic antibodies from recognizing its antigenic regions. Indeed, pre-enzymatic digestion of tissue samples for de-glycosylation improves the binding affinity of PD-L1 antibodies and its signal intensity, which allows for more accurate quantification of PD-L1 expression to guide the therapeutic efficacy of PD-1/PD-L1 inhibitors [190]. Functionally, PD-L1 glycosylation enhances its stability via antagonizing interactions with glycogen synthase kinase 3β (GSK3β) and related phosphorylation-dependent proteasome degradation [191]. N-linked glycosylation is also critical for its suppression of T cell immunity via interaction with its cognate receptor programmed cell death protein 1 (PD-1) [191, 192]. Collectively, these studies highlight de-glycosylation of PD-L1 as not only a biomarker, but also a promising therapeutic target for cancer immunotherapy.

## Conclusion and outlook

The field of cancer glycoproteomic biomarker studies is evolving rapidly with encouraging discoveries in cancer screening/early diagnosis, therapeutic prediction, and monitoring, among others. While these glycoproteomic signatures represent a promising possibility to serve as biomarkers, the field still faces challenges. First, it is still not fully clear if aberrant protein glycosylation patterns are unique to specific cancer types, pan-cancers or even more generalizable to other non-malignant pathologies including atypical hyperplasia, inflammation, infection, and other benign diseases. We need to better understand the association of the glycoprotein biology and cancer and other pathological processes. Further research is necessary to investigate the regulatory mechanisms underlying glycosylation sites and their biological consequences. Also, the role of neoplasia-induced hepatic reprogramming in the biomarker specificity remains to be determined. Secondly, we need to continue to advance with better technology and bioinformatics tools for ultrasensitive glycoproteomics to depict an in-depth and precise landscape of cancer. The development of small molecules or chip-based glycospecific techniques may also be necessary for their application in clinical settings given the poor affinity and imprecise specificities of lectin or antibody-based assays for occasional glycan epitopes. Finally, glycoproteomic biomarkers need to be translated into cancer care, which involves the leap from the proof of concept of retrospective small cohort studies to perspective and independent validation in both well-designed clinically investigations and real-world studies. Eventually, it needs to integrate with all clinical and pathological presentations, advanced imaging, and other tissue or liquid based omics, to provide a comprehensive but unique profile for precision care in cancer and risk management, diagnosis, and treatment.

In conclusion, years of advancing of glycoproteomic analyzing tools and algorithms, cancer biology and medicine, presents an emerging opportunity for scientists, physicians, industry and all involved, to work together towards further biomarker discovery and development, and ultimately to change the outcomes for cancer patients.
